# The effects of amyloidosis and aging on glutamatergic and GABAergic synapses, and interneurons in the barrel cortex and non-neocortical brain regions

**DOI:** 10.3389/fnana.2025.1526962

**Published:** 2025-02-12

**Authors:** Tao Qu

**Affiliations:** ^1^Molecular Neuroplasticity, German Center for Neurodegenerative Diseases (DZNE), Magdeburg, Germany; ^2^Medical Faculty, Otto-von-Guericke University, Magdeburg, Germany

**Keywords:** AD, aging, PV, SST, barrel cortex, non-neocortical brain regions

## Abstract

Previous studies on changes in the distribution of GABAergic interneurons and excitation/inhibition (E/I) balance in Alzheimer’s disease (AD) and aging were mainly conducted in the neocortex and hippocampus. However, the limbic system is the primary and crucial location for AD progression. Therefore, in this study, we utilized AD and aging mouse models to investigate the E/I balance and the distribution of parvalbumin (PV)- and somatostatin (SST)-expressing cells in S1BF (barrel field of primary somatosensory cortex, barrel cortex), CA1 hippocampal area and brain regions beyond the neocortex and hippocampus, including retrosplenial cortex (RSC, which is composed of RSG and RSA), piriform cortex (Pir), amygdala (BMA), and hypothalamus (DM). We discovered that amyloidosis may disrupt the alignment of excitatory pre- and postsynaptic quantities. Amyloidosis reduces the quantity of synapses and SST cells, but does not impact the counts of PV cells. By contrast, aging is linked to a decline in synapses, I/E ratios, SST and PV cells. Amyloidosis affects the S1BF and BMA, while aging may harm all studied regions, including the S1BF, RSC, hippocampus, Pir, BMA, and DM. Aging mostly affects synapses and I/E ratios in Pir, BMA, and DM, and PV and SST interneurons in the hippocampus.

## Introduction

Large amounts of abnormal expression of amyloid beta (Aβ), hyperphosphorylated tau (pTau) protein (Polanco et al., 2018), and loss of neurons and synapses (Crimins et al., 2013) are notable molecular features of AD. The amyloid cascade hypothesis of AD proposes that the aberrant production of Aβ is the first step in triggering the pathophysiological cascade that ultimately leads to AD (Tanzi and Bertram, 2005). Cerebrospinal fluid studies have shown that Aβ42 peptide abnormalities occur 1–20 years before the onset of AD symptoms (Bateman et al., 2012, Jia et al., 2024). As a model of AD, we used the 5xFAD mouse line with the C57BL6J genetic background, which carries five familial Alzheimer’s disease (FAD) mutations in APP and PS1 genes, resulting in overexpression of Aβ42 and rapid accumulation of Aβ plaques in the brain (Holly et al., 2006). In this study, we seek to compare the effects of aging and amyloidosis in adult 5xFAD mice in terms of alterations in the density of inhibitory and excitatory synapses, and densities of PV and SST interneurons in various brain regions.

As cortical state and excitatory input levels fluctuate, E/I balance changes accordingly. The proper functioning of brain activities relies on the maintenance of E/I balance. Senescence and a variety of neurodegenerative diseases are accompanied by E/I balance disorders and E/I ratio increases (Tran et al., 2019, Olde Engberink et al., 2023). Through the comparison of regional I/E values, we plan to study whether there are differences in E/I balance across different brain regions and different mouse models.

GABAergic interneurons, named for the release of the neurotransmitter gamma-aminobutyric acid (GABA), make up about 10-20% of cortical neurons (Xu et al., 2010). PVs, together with SST and vasoactive intestinal polypeptide (VIP), form three non-overlapping populations, accounting for about 85% of the neocortex interneurons (Karnani et al., 2016). Aging mainly affects the prefrontal cortex and relatively does not affect the limbic area (Zanto and Gazzaley, 2019). By comparison, AD mainly affects the limbic system (Hopper and Vogel, 1976), including the hypothalamus (Ishii and Iadecola, 2015), amygdala (Vereecken et al., 1994), thalamus (Forno et al., 2023), mammillary body (Tsivilis et al., 2008), and hippocampus (Villemagne et al., 2013, Rao et al., 2022). Based on this, we expect to describe the regional variability in research parameters and to compare the differences in the spatial distribution of PV cells and SST cells within the barrel cortex and areas beyond the neocortex in the mouse brain.

Different GABAergic neuron subtypes play different roles in E/I balance and different cerebral cortices. The most important source of inhibitory signaling throughout the cortex is parvalbumin (PV) interneuron (Terstege and Epp, 2023). A large number of studies have shown that the prefrontal cortex and its E/I balance are crucial for the regulation of cognitive behavior, and PV interneurons play an indispensable role in this (Staff and Spruston, 2003). PV interneurons can control the peak time of adjacent excitatory neurons, which is pivotal for maintaining proper E/I balance and regulating the functional connectivity of large brain networks (Bartholome et al., 2020). By comparing the correlations between PV, SST, and I/E, we try to explore which type of interneurons is more closely related to E/I balance in the studied mouse groups.

## Materials and methods

### Animals

In this study, we used male and female mice from 5xFAD transgenic mouse line (B6.Cg-Tg(APPSwFlLon, PSEN1*M146L*L286V)6799Vas/Mmjax with C57BL6J genetic background from the Jackson Laboratory). This mouse line was maintained by crossing of WT and heterozygous mice. We used 5 adult wild-type (WT) mice as control group, 6–10 month-old (it is equivalent to 30–40 year-old in humans (Flurkey et al., 2007)); 5 adult 5xFAD mice, 6–10 month-old; and 4 aged WT mice, 18–22 month-old (it is equivalent to 56–66 year-old in humans). All mice were acquired from the DZNE animal facility. The mice were housed under standard conditions of temperature (22 ± 2°C), humidity (60 ± 10%), and a 12-h light/dark cycle. The mice were provided with food and water ad libitum.

### Tissue preparation

All treatments and behavioral procedures were conducted in accordance with animal research ethics standards defined by German law and approved by the Ethical Committee on Animal Health and Care of the State of Saxony-Anhalt. To obtain the required samples, the mice were subjected to euthanasia. Anesthetized mice (ketamine/xylazine; 90 mg/kg and 18 mg/kg body weight in 0.9% NaCl solution) were transcardially perfused with ice-cold PBS followed by 4% paraformaldehyde (PFA) in 0.1 M phosphate buffer, pH 7.4 (PB). After mice were subjected to cervical dislocation, the brains were carefully dissected and fixed in 4% PFA in PBS for a period of 24 h, after which they were transferred to a 30% sucrose in PBS for cryoprotection. The brains were then sectioned at a thickness of 40 μm using a cryostat microtome (Leica, 1950) and collected in an ice-cold cryoprotectant solution (ethylene glycol-based: 30% ethylene glycol, 30% glycerol, 10% 0.2 M sodium phosphate buffer pH 7.4, in ddH_2_O), ensuring optimal preservation for subsequent analysis.

### Immunohistochemistry

Slices were washed in PBS with 0.5% Triton X-100, and blocked in 10% normal goat serum in PBS. Sections were incubated with primary antibodies 2 nights (for SST it’s 4 nights) at 4°C, incubated in secondary antibodies for 4 h at room temperature. Concentrations of antibodies were as follows: chicken anti-VGLUT1 (dilution 1:500, Synaptic Systems Cat# 135 316, RRID:AB_2619822), chicken anti-VGLUT2 (dilution 1:500, Synaptic Systems Cat# 135 416, RRID:AB_2619824), guinea pig anti-Homer1 (dilution 1:500, Synaptic Systems Cat# 160 004, RRID:AB_10549720), guinea pig anti-VGAT (dilution 1:500, Synaptic Systems Cat# 131 004, RRID:AB_887873), mouse anti-gephyrin (dilution 1:500, Synaptic Systems Cat# 147 011, RRID:AB_887717), rat anti-SST (Somatostatin) (dilution 1:200, Millipore Cat# MAB354, RRID:AB_2255365), rabbit anti- VIP (Vasoactive Intestinal Peptide) (dilution 1:500, ImmunoStar Cat# 20077, RRID:AB_572270), chicken anti-PV (Parvalbumin) (dilution 1:1,000, Synaptic Systems Cat# 195 006, RRID:AB_2619887), goat anti-chicken Alexa Fluor 488 (dilution 1:500, Invitrogen, A11039), goat anti-chicken Alexa Fluor 647 (dilution 1:1,000, Invitrogen, A21449), goat anti-rabbit Alexa Fluor 546 (dilution 1:500, Invitrogen, A11035), goat anti-rabbit Alexa Fluor 647 (dilution 1:500, Invitrogen, A21245), goat anti-rat Alexa Fluor 488 (dilution 1:200, Invitrogen, A11006), goat anti-rat Alexa Fluor 546 (dilution 1:200, Invitrogen, A11081), goat anti-mouse Alexa Fluor 488 (dilution 1:500, Invitrogen, A11029), goat anti-guinea pig Alexa Fluor 546 (dilution 1:500, Invitrogen, A11074). Finally, nuclei were counterstained with DAPI (dilution 1:400, Invitrogen, D1306), and slides were mounted with permanent mounting medium (Fluoromount™ Aqueous Mounting Medium) for microscopic evaluation.

### Confocal microscopy and image processing

**FIGURE 1:**
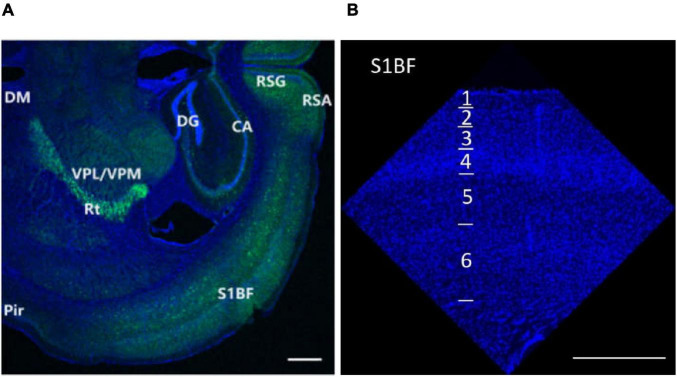
Research regions of all brain **(A)** and S1BF **(B)** in the mouse brain. The figures are high-resolution images of the brain **(A)** and S1BF **(B)**. The figures consist of DAPI-stained nuclei (blue dots) and PV cells (green dots). According to the description in “The mouse brain in stereotaxic coordinates” (Paxinos and Watson, 2008) and “The Allen Mouse Brain Atlas” (Allen institute), we determined the locations and abbreviations of mouse brain reigions. We performed immunohistochemical staining in plate bregma -1.4 mm of the mouse brain (**A,B**, Figures 2, 10, 11, 12). We conducted research of synapse connection, SST+ neurons, VIP+ neurons, and PV+ neurons in following areas, from L1 to L6 of S1BF (**A,B,** Figures 10, 11, 12), L1/2/3/5/6 of RSG and RSA, Rad/Py/Or of CA1/2/3, PoDG/GrDG, the reticular thalamus (Rt), ventral posteromedial nucleus of the thalamus (VPM), L1/2/3 of the piriform cortex (Pir), the amygdala (BMA), and the hypothalamus (DM) (**A,B**, Figures 11, 12). As shown in **(B)** and Figures 10, 11, 12, L1 to L6 of S1BF are distinguished by DAPI staining. L1 is a layer with sparse cells. L4 has the highest nucleus density. L2/3 is located between L1 and L4. The nuclear density falls substantially below L4, reaching L5. Further down is the layer 6, where the nuclear density increases significantly. The fringes below L6 are different from L6. Scale bars = 500 μm **(A,B)**.

Research regions are shown in Figure 1. Images were captured by confocal laser scanning microscopy (Zeiss LSM 700) and a Zeiss Axio Imager 2. For synapses, imaging was done with the EC Plan-Apochromat, 63 × /1.40 oil M27 objective (8-bit, 10 optical sections, 0.30 μm intervals between sections, 512 × 512 pixels, the pixel size of 0.07μm, and the scan zoom of 3.0). For SST, VIP, and PV cells, imaging was done with the EC Plan-Neofluar, 20 × /0.50 M27 objective (8-bit, 20 optical sections, 1.40 μm intervals between sections, 1024 × 1024 pixels, the pixel size of 0.63 μm, and the scan zoom of 0.50).

**FIGURE 2:**
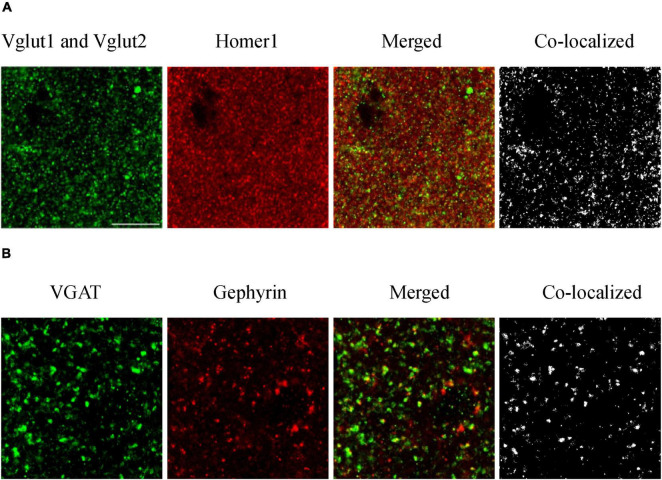
Immunofluorescence staining of excitatory **(A)** and inhibitory **(B)** synapses. To evaluate how amyloidosis and aging contribute to synaptic formation, we performed immunohistochemical analyses of excitatory synaptic markers VGlut1, VGlut2, and Homer1 and inhibitory synaptic markers VGAT and gephyrin in the mouse brain (Zander et al., 2010, Serrano et al., 2022). We conducted high-resolution confocal microscopy to examine excitatory synapses defined by co-localized VGlut1/VGlut2 and Homer1 immunopositive puncta and inhibitory synapses defined by co-localized VGAT and gephyrin puncta. Excitatory synaptic markers: Vglut1 and Vglut2 (pre), Homer1 (post); inhibitory synaptic markers: VGAT (pre) and Gephyrin (post). Synapses are composed of colocalized presynaptic and postsynaptic compartments. **(A)** S1BF-L1 of adult WT mice, and **(B)** S1BF-L5 of aged WT mice. Scale bar = 10 μm.

For synaptic density analysis, we conducted high-resolution confocal microscopy to examine excitatory synapses defined by co-localized VGlut1/VGlut2 and Homer1 immunopositive puncta, and inhibitory synapses defined by co-localized VGAT (vesicular GABA transporter) and gephyrin puncta (Figure 2). We utilized Fiji ImageJ (NIH) and Synapse Counter plug-in for ImageJ (Dzyubenko et al., 2016) to collect synapse data. Four brain slices were observed for each of the three mouse groups (one brain slice from each mouse and four mice representing each group). Four values were obtained from each brain region in each slice and then averaged. Four values were obtained from each group of mice.

Regarding the VIP, SST, and PV cells, four slices were analyzed for each of the three mouse groups (one brain slice from each mouse and four mice from each group). Two regions of interest (ROIs) from each brain region were analyzed and the derived values were averaged. Four values were obtained from each mouse group, except for the S1BF-L4 region of VIP where only 2 or 3 data were obtained due to imperfect staining. The three adjacent layers of 2.8μm vertical axis with the largest cell counts were projected onto a surface, and the total number of interneurons on the surface was calculated and compared. ZEN 3.5 (blue edition) was used to manually quantify the cells in each image.

Statistical significance was assessed by analyzing the numerical differences in several brain regions across different groups of mice.

### Statistics

**FIGURE 3:**
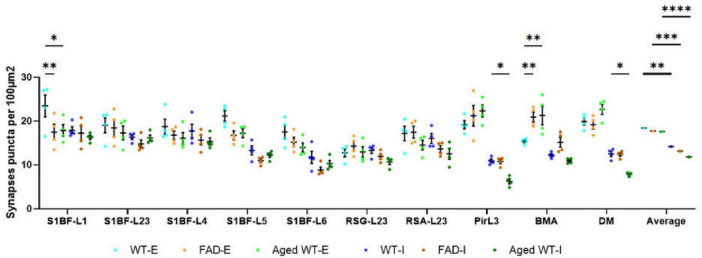
Synaptic puncta per 100 μm^2^. A RM Two-Way ANOVA with Holm-Sidak post-hoc analysis was used to compare data between different groups of mice in each brain region or to compare the average data across all studied regions between different groups of mice. We found that compared with adult WT controls, the excitatory synapses of adult 5xFAD mice are significantly reduced in S1BF-L1, while those of BMA are significantly increased. There is no significant difference in the number of inhibitory synapses between adult 5xFAD mice and adult WT controls. Compared with adult WT controls, aged WT mice have significantly reduced excitatory synapses in S1BF-L1, but significantly increased excitatory synapses in BMA. The number of inhibitory synapses at Pir, DM are significantly reduced in aged WT mice (*p < 0.05, **p < 0.01). A RM Two-Way ANOVA with Holm-Sidak post-hoc analysis was used to compare the average data across all studied regions between excitatory and inhibitory synapses in each mouse group. The number of excitatory synapses is significantly higher than that of inhibitory synapses in each mouse group (**p < 0.01, ***p < 0.001, and ****p < 0.0001). WT-E, excitatory synapse of adult wild-type controls. FAD-E, excitatory synapse of adult 5xFAD mice. Aged WT-E, excitatory synapse of aged wild-type mice. WT-I, inhibitory synapse of adult wild-type controls. FAD-I, inhibitory synapse of adult 5xFAD mice. Aged WT-I, inhibitory synapse of aged wild-type mice.

**FIGURE 4:**
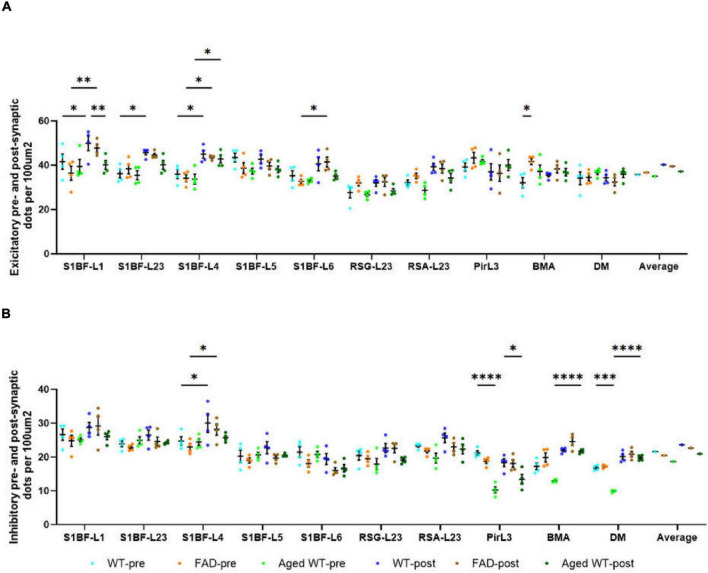
The number of pre- and post-synaptic puncta in excitatory **(A)** and inhibitory synapses **(B)** per 100 μ m^2^. A RM Two-Way ANOVA with Holm-Sidak *post-hoc* analysis was used to compare data between pre- and postsynapses or to compare data between three groups of mice. Details of statistical significance are in the results. WT-pre, pre-synapse of adult wild-type mice. FAD-pre, pre-synapse of adult 5xFAD mice. Aged WT-pre, pre-synapse of aged wild-type mice. WT-post, post-synapse of adult wild-type mice. FAD-post, post-synapse of adult 5xFAD mice. Aged WT-post, post-synapse of aged wild-type mice.

**FIGURE 5:**
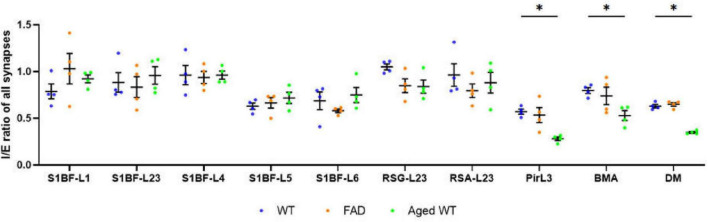
Ratio of inhibitory to excitatory synaptic counts (I/E) of all brain regions. A RM Two-Way ANOVA with Holm-Sidak *post-hoc* analysis was used to compare data between three groups of mice. Previous studies revealed disease-associated decline of I/E in different brain regions (Tran et al., 2019, Lauterborn et al., 2021, Olde Engberink et al., 2023, Scaduto et al., 2023). We observed a significant decrease in I/E in the Pir, BMA, and DM of aged WT mice (*p < 0.05). A significant difference [*p* < 0.0001, *F*(9, 27) = 6.644 for adult WT controls, *p* < 0.0001, *F*(9, 27) = 14.94 for aged WT mice, and *p* < 0.001, *F*(9, 27) = 5.346 for adult 5xFAD mice] is seen in the I/E ratios among different brain regions within each group of mice using RM One-Way ANOVA. We can see that the regions with the highest I/E values are located in L1 to L4 of S1BF, RSC, and BMA of the three groups of mice.

**FIGURE 6:**
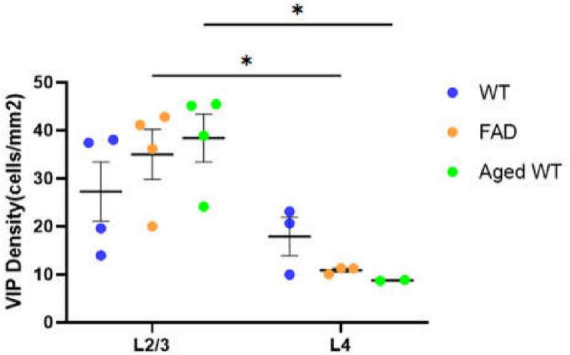
Laminar density of VIP interneurons in S1BF. *P < 0.05 between S1BFL2/3 and L4 of the VIP cell density both in adult 5xFAD mice and aged WT mice using mixed-effects Two-Way ANOVA with Holm-Sidak *post-hoc* analysis.

**FIGURE 7:**
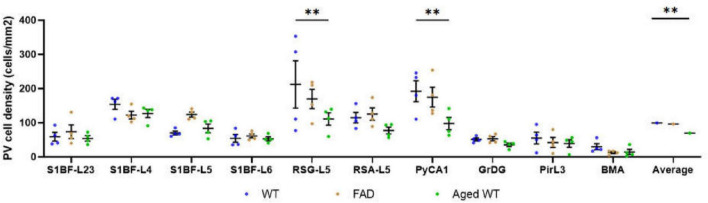
PV cell density (cells/mm^2^). A RM Two-Way ANOVA with Holm-Sidak *post-hoc* analysis was used to compare data between different groups of mice in each brain region or to compare the average data of all studied regions between different groups of mice. The spatial density distribution of PV interneurons is different among the three groups of mice. Compared with WT controls, the number of PV cells in RSG and CA1 regions of aged WT mice is significantly reduced (**p < 0.01). The average quantity of PV cells in aged WT mice is lower than that in adult WT controls (**p < 0.01), while the difference between adult WT controls and adult 5xFAD mice is not significant.

**FIGURE 8:**
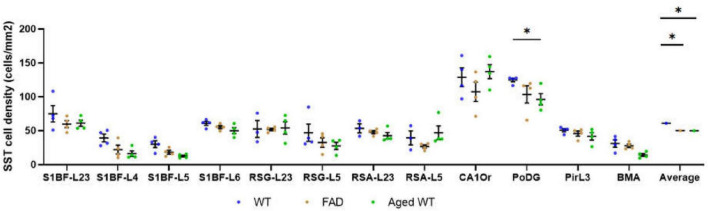
SST cell density (cells/mm^2^). The spatial density distribution of SST interneurons varies among the three groups of mice. A mixed-effects Two-Way ANOVA with Holm-Sidak post-hoc analysis was used to compare data between different groups of mice in each brain region or to compare the average data of all studied regions between different groups of mice. Compared with WT controls, the number of SST cells in aged WT mice decreased significantly in the PoDG (*p < 0.05). The average quantity of SST cells in adult 5xFAD mice and aged WT mice is lower than that in adult WT controls (*p* < 0.001). A significant difference is seen in SST cell density across several brain locations within each group of mice [*p* < 0.0001, *F*-values are *F*(11, 30) = 17.28, *F*(11, 33) = 19.27, and *F*(11, 33) = 29.74, respectively] using RM or mixed-effects One-Way ANOVA with Holm-Sidak *post-hoc* analysis. In all three mouse groups, the brain regions with the highest density of SST cells are the same, L2/3/6 in S1BF, RSC-L2/3, CAOr and PoDG in the rest of the study regions (in this figure, Figure 12).

Graphpad Prism 8.0 was used for plotting and executing statistical analysis. Error bars in Figures 3–5, 6, 7, 8 represent the mean ± SEM values. *p < 0.05, **p < 0.01, ***p < 0.001, and ****p < 0.0001 indicate statistically significant differences. A RM One-Way ANOVA was employed to evaluate the summary p value on synapse density, I/E ratios, or interneuron density across several brain areas in each group of mice. A RM Two-Way ANOVA with Holm-Sidak *post-hoc* analysis was used to compare data between pre- and postsynapses, to compare synapse density between excitatory and inhibitory synapses, or to compare data between three groups of mice. Mixed-effects One-Way or Two-Way ANOVA with Holm-Sidak *post-hoc* analysis were used to evaluate data containing missing values. A Three-Way ANOVA with Holm-Sidak *post-hoc* analysis was used to compare the mean synapse densities of both excitatory and inhibitory synapses across all studied regions between different groups of mice.

Pearson’s correlation coefficient (r) is used to study the correlation between two variables. The greater the absolute value of r (| r|), the stronger the correlation. With the used number of samples, a correlation coefficient 0 means no correlation, between 0 and 0.5 suggests a weak correlation, between 0.5 and 0.8 suggests a moderate correlation, and ≥ 0.8 indicates a strong correlation. *p* < 0.05 or *p* < 0.01 indicates the significant linear correlation between the two variables.

## Results

Notably, our study highlights the considerable roles of amyloidosis and aging in synaptic transformation, resulting in impaired synaptic connections and rearranged synaptic distribution in different groups of mice. Specifically, we observed statistically significant differences in synaptic properties (excitatory or inhibitory synapses) and synapse counts in various brain regions among three groups of mice. Furthermore, we have validated those synaptic regions with the highest or lowest number of synapses in adult 5xFAD and aged WT mice had undergone relocation compared to adult WT controls (Figure 3).

A RM One-Way ANOVA with Holm-Sidak *post-hoc* analysis was used to compare the synapse density between different brain regions of each mouse group. There are significant differences for the synapse density between different brain regions in each mouse group. Specifically, for excitatory synapses, the AVOVA values are *p* < 0.001, *F*(9, 27) = 5.697 in adult WT controls, *p* < 0.001, *F*(9, 27) = 6.119 in aged WT mice, and *p* < 0.01, *F*(9, 27) = 3.721 in adult 5xFAD mice; for inhibitory synapses, *p* < 0.0001 in adult WT controls [*F*(9, 27) = 10.61], adult 5xFAD mice [*F*(9, 27) = 13.74], and aged WT mice [*F*(9, 27) = 24.88].

As shown in Figure 3, in adult WT controls, the highest density of excitatory synapses is in S1BFL1 (23.4 ± 2.5/100 μm^2^) and L5 (21.1 ± 1.2/100 μm^2^), but in adult 5xFAD and aged WT mice, they are in PirL3 (21.2 ± 2.4/100 μm^2^, 22.3 ± 1.4/100 μm^2^), BMA (20.9 ± 1.1/100 μm^2^, 21.3 ± 2.1/100 μm^2^), and DM (19.1 ± 1.0/100 μm^2^, 22.7 ± 1.1/100 μm^2^). RSGL2/3 has the lowest density of excitatory synapses amongst all the brain regions studied (adult WT controls 12.7 ± 0.9/100 μm^2^, adult 5xFAD mice 14.3 ± 1.1/100 μm^2^, and aged WT mice 12.9 ± 1.2/100 μm^2^). The highest density of inhibitory synapses of the three groups of mice are observed in L1, L2/3, and L4 of S1BF (adult WT controls: 17.9 ± 0.8/100 μm^2^, 16.3 ± 0.5/100 μm^2^, and 17.7 ± 1.6/100 μm^2^; adult 5xFAD mice: 17.2 ± 1.6/100 μm^2^, 14.8 ± 0.9/100 μm^2^, and 15.7 ± 1.1/100 μm^2^; aged WT mice: 16.3 ± 0.5/100 μm^2^, 16.2 ± 0.6/100 μm^2^, and 15.3 ± 0.8/100 μm^2^). The regions with the lowest density of inhibitory synapses are S1BF-L6 and PirL3 for adult WT (11.7 ± 1.4/100 μm^2^ and 10.9 ± 0.4/100 μm^2^) and 5xFAD mice (8.8 ± 0.7/100 μm^2^ and 10.8 ± 0.5/100 μm^2^), while PirL3 and DM are the ones with the lowest density of inhibitory synapses for aged WT mice (6.3 ± 0.6/100 μm^2^ and 7.9 ± 0.2/100 μm^2^).

There is no significant difference between three groups of mice in the mean synapse density of excitatory or inhibitory synapses across all studied regions (Figure 3, *P* > 0.05, comparing the average data across all studied regions between different groups of mice using RM Two-Way ANOVA with Holm-Sidak *post-hoc* analysis). However, adult and aged WT mice are different in the mean total synaptic density (combining both excitatory and inhibitory synapses) across all studied regions (*P* < 0.05, *F*(1, 6) = 7.506 using three-Way ANOVA with Holm-Sidak *post-hoc* analysis].

The ratios of the mean values of RSA-L2/3 to RSG-L2/3 excitatory synapses in adult WT, adult 5xFAD, and aged WT mice are 1.35, 1.22, and 1.12, respectively. Meanwhile, the ratios of the mean values of RSA-L2/3 to RSG-L2/3 inhibitory synapses are 1.21, 1.14, and 1.14 in adult WT, adult FAD, and aged WT mice, respectively.

There are statistical differences between the density of excitatory pre- and postsynaptic markers in such brain regions: S1BF-L1, L2/3, and L4 of adult WT controls; S1BF-L1, L4, and L6 of adult 5xFAD mice; S1BF-L4 of aged WT mice (Figure 4A, *p < 0.05, **p < 0.01). There are statistical differences between the density of excitatory presynapses of adult WT controls and adult 5xFAD mice in BMA (Figure 4A, *p < 0.05). This suggests an increase in Vglut1/2-immunopositive presynapses in BMA in adult 5xFAD mice compared to adult WT controls. There are statistical differences between the density of excitatory postsynapses of adult WT controls and aged WT mice in S1BF-L1 (Figure 4A, **p < 0.01). This suggests a decrease in Homer1-immunopositive postsynapses in S1BF-L1 in aged WT mice group compared to adult WT controls.

The densities of inhibitory pre- and postsynaptic puncta exhibit statistical differences in the following regions: S1BF-L4 of adult WT controls and adult 5xFAD mice, and BMA and DM of aged WT mice (Figure 4B, *p < 0.05, ****p < 0.0001). The densities of inhibitory pre- and postsynaptic puncta in brain areas RSC and Pir do not exhibit any statistically significant changes. Statistical disparities exist across three distinct mouse groups in terms of inhibitory pre- or post-synapses within certain regions. A statistically significant difference exists in the density of pre-synapses between adult WT controls and aged WT mice in Pir and DM (Figure 4B, ***p < 0.001, ****p < 0.0001). This difference implies a reduction in the density of VGAT-immunopositive presynapses in Pir and DM in aged WT mice group as compared to their adult counterparts. A statistically significant difference is found in the density of post-synapses between adult WT controls and aged WT mice in Pir (Figure 4B, *p < 0.05). This suggests that there is a reduction in gephyrin-immunopositive postsynapses in Pir in aged WT mice group as compared to their adult counterparts.

**FIGURE 9:**
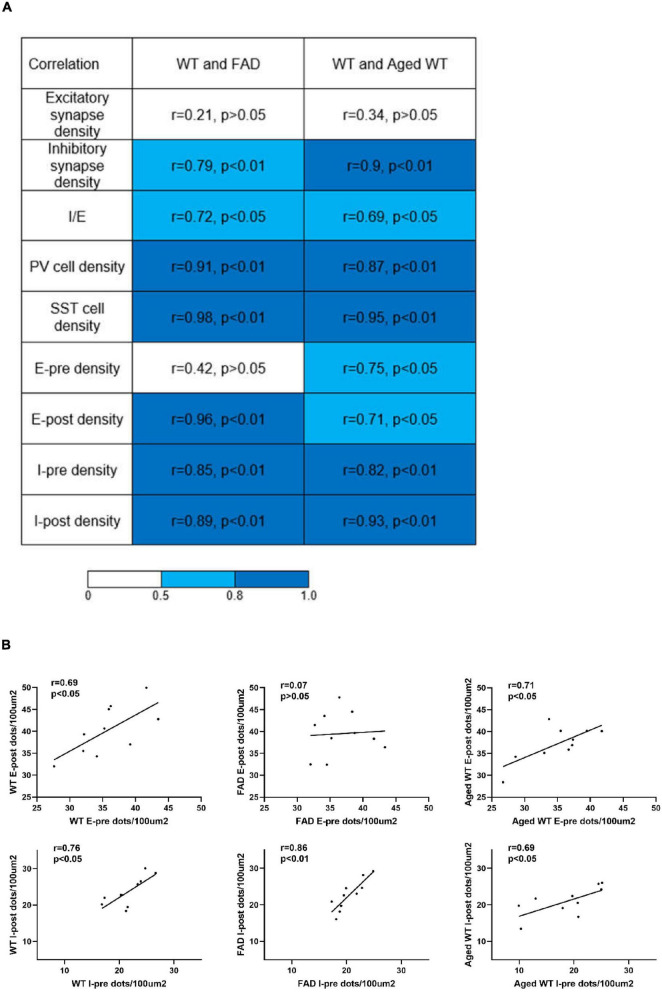
Analysis of correlations between parameters measured in adult WT, adult 5xFAD and aged WT mice across brain regions. **(A)** Pearson’s correlation coefficients and the corresponding p-values of synapses, I/E, PV cells, and SST cells, pre- and post-synapses in excitatory and inhibitory synapses between adult WT, adult 5xFAD and aged WT mice. **(B)** Pearson’s correlation coefficients and corresponding p-values between pre- and post-synapses in each mouse group. E-pre, excitatory presynapses. E-post, excitatory postsynapses. I-pre, inhibitory presynapses. I-post, inhibitory postsynapses. The mean of the four values for each brain location was computed, and the values from ten or twelve (SST cells) brain locations were compared among different mouse groups to establish correlations of the research targets (synapses, I/E, or interneurons) among different mouse groups. The | r| value grows as the color transitions from light blue to dark blue. A correlation coefficient between 0.5 and 0.8 suggests a moderate correlation, and ≥ 0.8 indicates a strong correlation. *P* < 0.05 or *P* < 0.01 indicates the significant linear correlation between the two variables.

The AD and aging mouse models are highly correlated with adult WT controls in both pre- and post-synapses, with the exception of the excitatory presynapses between adult 5xFAD mice and adult WT controls (Figure 9A, E-pre, E-post, I-pre, and I-post). In addition, there is a lack of statistical distinction between the mouse models and the control group in the majority of research regions (Figures 4A,B).

When comparing excitatory and inhibitory synapses that are colocalized by pre- and post-synaptic markers (Figure 2), it is observed that the density of excitatory synapses exhibits a weak correlation across various mouse groups. Conversely, the density of inhibitory synapses demonstrates a moderate or strong correlation among different mouse types (Figure 9A, excitatory and inhibitory synapse density). In the context of aged WT mice, it has been observed that there is a certain proportion of excitatory or inhibitory synapses that are statistically different from adult WT controls. There is no significant difference observed in the inhibitory synapses between adult 5xFAD mice and adult WT controls (Figure 3).

**FIGURE 10:**
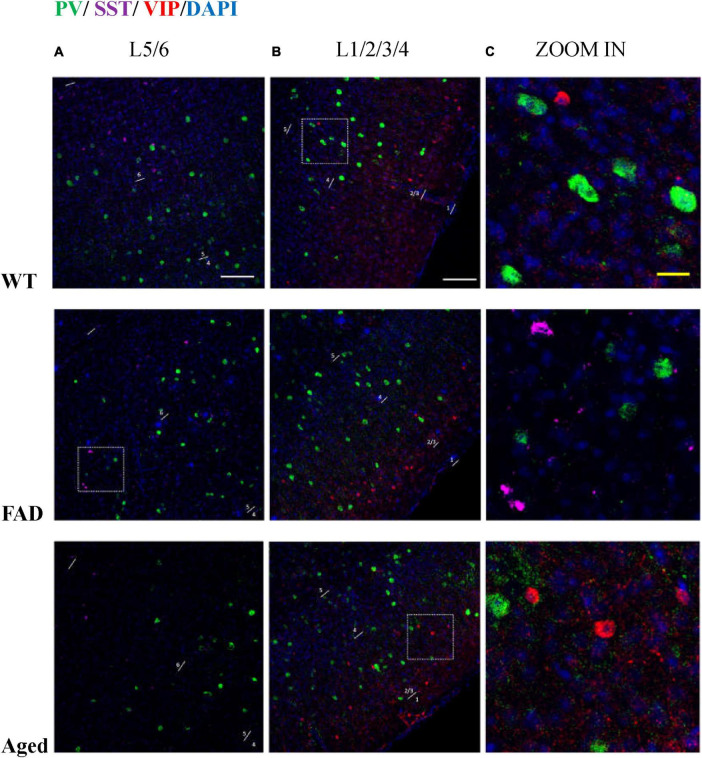
PV, SST, and VIP interneurons are scattered in different areas in S1BF. **(A)** The distribution of PV interneurons (green) and SST interneurons (purple) in L5/6 of S1BF. The blue color represents DAPI staining. **(B)** The distribution of PV interneurons (green) and VIP interneurons (red) in L1/2/3/4 of S1BF. **(C)** Zoomed image of white dotted box area in **(A,B)**. Scale bars: 100 μm **(A,B)** and 20 μm **(C)**. The regions with the highest density of PV, SST, and VIP cells are different in S1BF: L4/5 for PV cells **(A,B)**, L2/3 and L6 for SST cells (**A**, Figure 12), and L2/3 for VIP cells (**B**, Figure 6). In S1BF region, the cell density of SST cells in L6 is higher than that in L5 (**A**, Figures 8, 12). The density of VIP cells in L2/3 is greater than that in L4 (Rudy et al., 2011), of which the upper half of L2/3, namely L2, is the densest area (**B**, Figure 6). There is no colocalization between PV, SST, and VIP cells **(C)**. Meanwhile, the three cell distribution levels are different in three-dimensional space. For example, in the zoomed images **(C)**, the PV cells surrounding VIP and SST cells (**C**, row 2, 3) appeared less defined compared to other PV cells (**C**, row 1), indicating that PV cell cores exist in adjacent layers. The average distances between the core of VIP cells and PV cells are 3.68, 3.68, and 4.9 μm in three groups of mice, respectively. The average distances between the core of SST cells and PV cells are 3.33, 3.15, and 3.73 μm in three groups of mice, respectively.

**FIGURE 11:**
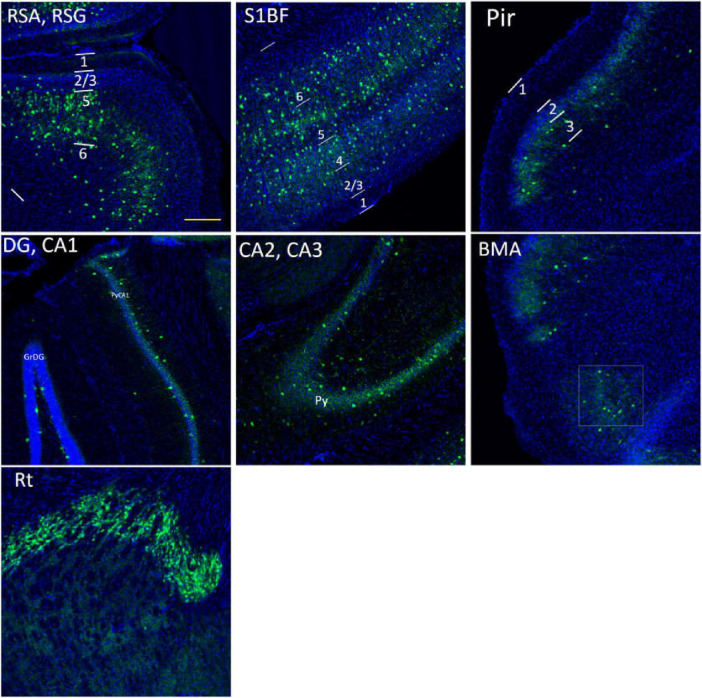
The distribution of PV cells (green color) in the S1BF, the hippocampus and other brain regions in adult WT controls. The blue color is DAPI staining. Following is the detailed description of PV cell distribution. RSG/RSA: mainly in L5, sparsely in L2/3, and absence in L1. S1BF: in L2/3/5/6a, and no distribution in L1/6b. Pir: sparsely in L3, and no distribution in L1/2. The hippocampus: concentrated in PyCA1/2/3, GrDG, and virtually undetectable in other layers. BMA, sparsely distributed. Rt, extremely densely distributed. There are no PV cells in DM area. Scale bar = 200 μm.

A significant difference [*p* < 0.001, *F*(9, 27) = 5.746 in adult WT controls, *p* < 0.0001, *F*(9, 27) = 11.61 in adult 5xFAD mice, and *p* < 0.0001, *F*(9, 27) = 8.999 in aged WT mice] is seen in the PV cell density among different brain regions within each group of mice using RM One-Way ANOVA with Holm-Sidak post-hoc analysis. In the three group of mice, the brain regions with the highest density of PV cells are the same: L4/5 in S1BF (Figures 7, 10A,B, 11), and RSG-L5 and PyCA in the rest of the studied regions (Figures 7, 11). The PV density ratio of RSG-L5 to RSA-L5 is 1.8, 1.4, and 1.4 in adult WT controls, adult 5xFAD mice, and aged WT mice, respectively. Additionally, the PV density ratio of PyCA1 to GrDG is 3.8, 3.3, and 2.7 in adult WT controls, adult 5xFAD mice, and aged WT mice, respectively.

For three groups of mice, the correlation among their excitatory synapse densities varied, whereas their inhibitory synapses are closely related (Figure 9A). The excitatory synapse number of adult WT controls is weakly correlated with that of other two groups of mice: *r* = 0.21 between WT-E and FAD-E, and *r* = 0.34 between WT-E and Aged WT-E. The inhibitory synapse readouts of three groups of mice are closely correlated: *r* = 0.79 between WT-I and FAD-I, *r* = 0.90 between WT-I and Aged WT-I. We found moderate correlations in the I/E ratios among adult WT, adult FAD, and aged WT mice (0.5 < *r* < 0.8, *p* < 0.05). We observed a strong correlation in PV cell density or SST cell density between adult WT, adult 5xFAD, and aged WT mice (*r* > 0.8, *p* < 0.01). PV cell density of adult WT controls is strongly correlated with that of adult 5xFAD mice (*r* = 0.91) and aged WT mice (*r* = 0.87). Similarly, SST cell density of adult WT controls is strongly correlated with that of adult 5xFAD mice (*r* = 0.98) and aged WT mice (*r* = 0.95). That means the densities of both PV and SST cell increase or decrease proportionally in different groups of mice.

## Discussion

### A higher region-specific susceptibility of excitatory synapses to aging and amyloidosis

Normally, Aβ is only expressed in small amounts in the brain, where it has neurotrophic effects on immature, undifferentiated neurons. In the brain of AD patients, the expression of Aβ is abnormal and greatly increased, and it has toxic effects on differentiated and mature neurons (Gouras et al., 2015). The amyloid hypothesis suggests that overexpressed Aβ is the trigger for all pathophysiological responses to AD (Glenner and Wong, 1984, Selkoe and Hardy, 2016). Oligo-Aβ42 is the most toxic among all Aβ species (Hu et al., 2014). 5xFAD mice are widely used because they have large amounts of Aβ42 that accumulate abnormally.

For three groups of mice, the correlations among their excitatory synapse densities are weak across brain regions, whereas their inhibitory synapses are closely related (Figure 9A). This suggests a higher region-specific susceptibility of excitatory synapses to aging and amyloidosis. Previous studies implied that Aβ oligomers contribute to synaptic shifts (Palop et al., 2007, Li and Selkoe, 2020, Walsh and Selkoe, 2020). Aβ42 oligomers induce overexpression of Vti1a, leading to high frequency spontaneous vesicular exocytosis associated with Glu, which is further accompanied by downregulation of VGLUT1, and eventually cause depletion of Glu content in vesicles and significantly reduced Glu release. Ultimately, this sequence of events contributes to neurodegeneration in the later stages of AD (Zott et al., 2019, Yang X.-K. et al., 2023).

The sites of the most excitatory synapses are L1/5 in adult WT, are PirL3, BMA, DM in adult 5xFAD and aged WT mice. These regions are closely related to cognitive function (Joyce et al., 2024), olfaction (Blazing and Franks, 2020), emotional response (Patin and Pause, 2015), endocrine system (Ishii and Iadecola, 2015), etc.

Statistical variances in synaptic density within the same brain region among different groups of mice suggest that amyloidosis impacts synaptic density in S1BF and BMA, while aging affects synaptic density in S1BF, Pir, BMA, and DM in the mouse brain (Figures 3, 4). Our findings support the idea that amyloidosis and senescence might remodel synaptic connections and synaptic distribution in mouse brains.

The excitatory synaptic ratio of RSA-L2/3 to RSG-L2/3 is 1.35, 1.22, and 1.12 in adult WT, adult 5xFAD, and aged WT mice, respectively. Meanwhile, the inhibitory synaptic ratio of RSA-L2/3 to RSG-L2/3 is 1.21, 1.14, and 1.14 in adult WT, adult FAD, and aged WT mice, respectively. Previous studies have shown that pyramidal neurons in layer 4/5 of RSG send lateral rami of ipsilateral and contralateral axons to layer 2/3 of RSA (Sugar et al., 2011). From our data, we can see that the number of synapses at the RSA-L2/3 site is relatively reduced in the adult 5xFAD and aged WT mice.

There are more excitatory synapses than inhibitory synapses, which aligns with a prior study indicating that there are more glutamatergic neurons than GABAergic neurons in the cerebral cortex (Rubenstein and Merzenich, 2003).

### Amyloidosis may disrupt the alignment of excitatory pre- and postsynaptic quantities across brain regions

Through a comparative analysis of pre- and post-synaptic densities within each mouse groups, it is shown that all synapses exhibit a moderate to high level of correlation, with the exception of the excitatory synapses in adult 5xFAD mice (Figure 9B). This finding aligns with a previous study that reported a match between pre- and postsynaptic structures (Kay et al., 2011). Importantly, we found that amyloidosis may disrupt the alignment of excitatory pre- and postsynaptic quantities. Aβ fibril may stimulate glial cells to overrelease excitatory amino acids, such as glutamate. At the same time, Aβ causes the mislocalization and internalization of the fast excitatory amino acid transporter (EAAT) and changes the clearance of glutamate (Scimemi et al., 2013). Glutamate is the main excitatory neurotransmitter in the brain (Orrego and Villanueva, 1993), and excitatory toxicity hypothesis refers to the frequent stimulation of neurons by excessive glutamate leading to neuronal dysfunction (Belov Kirdajova et al., 2020). This excitotoxicity eventually leads to neuronal calcium overload and has been linked to neurodegenerative diseases (Lipton and Rosenberg, 1994). There is a moderate or strong correlation between the densities of inhibitory pre- and post-synapses (Figure 9B, I-pre and I-post), and there are only a few regions where there are significant differences between them (Figure 4B, S1BF-L4, BMA and DM). In the RSC and Pir, there is no statistically significant difference between the pre- and post-synapses of excitatory or inhibitory synapses (Figures 4A,B).

### Brain regions L1–L4 of S1BF, RSC, and BMA exhibit the highest levels of inhibition compared to other brain regions

We found moderate correlations for I/E ratios among adult WT, adult 5xFAD, and aged WT mice (Figure 9A). Although the levels of cortical activities may fluctuate, the single pyramidal cell still maintains a balance of synaptic excitation and inhibition (E/I balance) (Okun and Lampl, 2008, Atallah and Scanziani, 2009). The mechanism behind this might be that as excitation increases, inhibition will increase correspondingly. E/I balance contributes indispensably to the cognitive functions like memory, learning, etc. (Froemke, 2015).

Here we can see that the regions with the highest I/E values are located in L1–L4 of S1BF, RSC, and BMA of the three groups of mice. Our synaptic studies in mouse models of AD, aging as well as WT controls had shown that brain regions S1BF, RSC, and BMA are of highest relative inhibition (Figure 5).

AD, schizophrenia, and autism are accompanied by an increased E/I ratio and a relative increase in excitability. However, the E/I balance of major depression shifted toward inhibition (Yang B. et al., 2023). In patients with depression, connectivity changes are frequently reported in several brain regions, including the amygdala, frontal cortex (Howard et al., 2019), anterior cingulate cortex, and ventral striatum (Pruessner et al., 2010, Otte et al., 2016, Roddy et al., 2021, Chai et al., 2023). Amygdala is involved in the processing of fear memory (Zhou et al., 2018, Chai et al., 2023). It can be seen that the regions with the strongest relative inhibition in 5xFAD and aged WT mice are highly coincident with the main lesion area of depression. So far, the correlation between depression and dementia is in dispute. Some literatures suggest that the two are related (Lutz et al., 2020, Elser et al., 2023), whilst others do not (Huang et al., 2020).

### Non-neocortical brain regions contribute the most to the fluctuation in I/E ratio in aged WT mice

Inhibitory variation leads to the E/I (excitation/inhibition) balance disorder (Francis, 2005; Le Roux et al., 2008). AD and aging are characterized by an E/I imbalance (Busche and Konnerth, 2016), a relative glutamatergic overexcitability (Lipton and Rosenberg, 1994; Reisberg et al., 2003; Francis, 2005), and a decrease in GABAergic activity (Busche and Konnerth, 2016). Non-neocortical brain regions contribute the most to the fluctuation in I/E ratio. Pir, BMA and DM in aged WT mice exhibit the largest drop in the I/E ratio together (Figure 5). The piriform cortex is an important part of the olfactory pathway, and its damage leads to olfactory disorders (Neville and Haberly, 2004; Attems et al., 2015). Researches have shown that olfactory dysfunction appears as an early symptom in normal elderly people (Schubert et al., 2012). Hypothalamic abnormalities are associated with memory impairment (Hamani et al., 2008).

### Aging affects the distribution of PV interneurons in the mouse brain, while amyloidosis does not

Notably, aging affects PV cells in the RSG and CA1 region of hippocampus apparently, but amyloidosis doesn’t show this effect (Figure 7). We can deduce that aging damages retrosplenial cortex and hippocampus of the mouse brain. RSC dysfunction is correlated with learning deterioration, and RSA neurons encode task-related dimensions across learning in mice (Sun et al., 2021). Furthermore, RSG (Sigwald et al., 2016; Tsai et al., 2022) or RSA (Pan et al., 2022) may play a predominant role in the recall of contextual fear memory. This discrepancy is also reflected in the average number of PV cells in the three types of mice. The average quantity of PV cells in aged WT mice is lower than that in adult WT controls, although the difference between adult WT controls and adult 5xFAD mice is not significant (Figure 7). This implies that aging affects the distribution of PV interneurons in the mouse brain, while amyloidosis does not. It is in line with the previous study in humans, which suggested that PV interneurons exhibited relative resistance to degeneration in AD patients (Ferrer et al., 1991).

### Both amyloidosis and aging affect the distribution of SST interneurons in the mouse brain

Compared with adult WT controls, the number of SST in PoDG is significantly decreased in aged WT mice (Figure 8). We can infer that aging damages the hippocampus of the mouse brain. The average quantity of SST cells in adult 5xFAD mice and aged WT mice is lower than that in adult WT controls. It indicates that both amyloidosis and aging affect the distribution of SST interneurons in the mouse brain. Previous studies have shown that the decline of SST in AD patients and AD mouse models is related to factors such as Aβ, ptau, APOE4 genes, etc. (Xu et al., 2020). However, decreased SST in elderly patients and mice may cause Aβ formation (Watamura et al., 2022).

### PV and SST interneurons are not overlapping in the hippocampus

Inhibitory interneurons mainly comprise PV, VIP, SST, and a few other interneurons (Kelsom and Lu, 2013; Yavorska and Wehr, 2016). Aβ causes inhibitory interneurons dysfunction (Zhang et al., 2017; Ranasinghe et al., 2022). Previous studies have shown that SST, VIP, and PV interneuronal markers have no co-localization in the cerebral cortex (Suzuki and Bekkers, 2010; Rudy et al., 2011), which echoes our study that PV, SST, VIP interneurons are scattered in different areas in S1BF (Figure 6).

**FIGURE 12:**
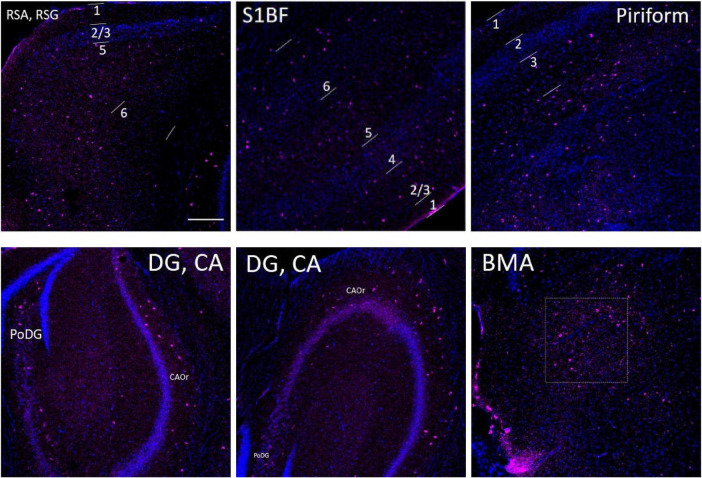
The distribution of SST cells (purple color) in the S1BF, the hippocampus and other brain regions in adult WT controls. The blue color represents DAPI staining. Following is the detailed description of SST cell distribution. RSG and RSA: mainly distributed in L2/3 and L5, but also in L1 and L6. Unlike PV density disparities at RSGL2/3 and RSGL5, there is little difference between SST cell density at RSGL2/3 and RSGL5. S1BF: mainly distributed in L2/3/6, also distributed in L4/5. Pir: mostly in L3, and no distribution in L1/2. The hippocampus: all evenly distributed along CA1/2/3Or all the way to PoDG. Not on PyCA or GrDG. BMA: sparsely distributed. There are no SST cells in hypothalamus area. Scale bar = 200 μm.

**FIGURE 13:**
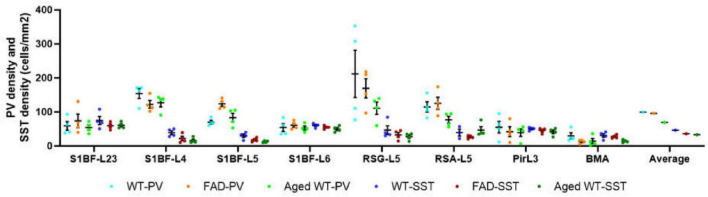
Comparison of PV cell density and SST cell density in each brain region (cells/mm^2^). In the S1BF region, layers 2/3 and 6 have the lowest distribution of PV cells but the highest distribution of SST cells, and they also have the most similar density of PV and SST cells. L4 and L5 have the highest PV cell density and the lowest SST cell density, with the largest difference in PV and SST cell densities among the six layers of S1BF. In RSG and RSA regions, PV and SST cell densities differ greatly. The densities of PV and SST cells in Pir and BMA are similar.

In the RSG/RSA region, PV cells are mainly distributed in L5 (Figures 7, 11), while SST cells are evenly distributed in L2/3 and L5 (Figures 8, 12). At the hippocampus, PV cells are mainly distributed in PyCA and GrDG, while SST cells are mainly distributed in CAOr and PoDG. PV and SST interneurons are scattered in different areas in the retrosplenial cortex and hippocampus, and these two populations are not overlapping in the hippocampus. In the S1BF region, layers 2/3 and 6 have the lowest distribution of PV cells but the highest distribution of SST cells, and they also have the most similar density of PV and SST cells (Figure 13). L4 and L5 have the highest PV cell density and the lowest SST cell density, with the largest difference in PV and SST cell densities among the six layers of S1BF.

We observed that there are no PV or SST cells in the hypothalamus in all three groups of mice.

### PV interneurons may more prominently regulate E/I balance

**FIGURE 14:**
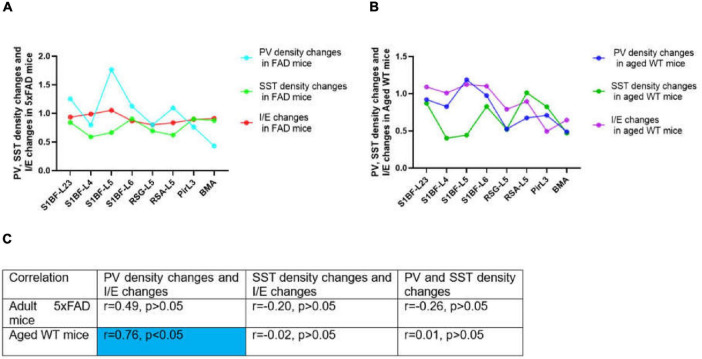
Correlations between PV or SST density changes and I/E changes across brain regions. In **(A,B)**, changes stand for the ratio of the value in adult 5xFAD or aged WT mice to the value in adult WT controls. The value was computed by the mean of the four values in each brain location. In both adult 5xFAD and aged WT mice, S1BF-L5 is the area where relative inhibition grow the most (**A,B**, I/E changes). Interestingly, in both adult 5xFAD and aged WT mice, S1BF-L5 is also the region with the highest increase in PV cells (**A,B**, PV density changes). In **(C)**, the | r| value grows as the color transitions from light blue to dark blue. The significance level for testing is α = 0.05. Previous studies have demonstrated a correlation between PV interneurons and E/I balance (Xue et al., 2014, Ferguson and Gao, 2018), although the extent of their interaction is still uncertain. We have completed investigation in multiple brain regions and found a weak correlation between the changing rate of PV interneurons density and I/E in adult 5xFAD mice (*r* = 0.49, *p* > 0.05), and a moderate correlation in aged WT mice (*r* = 0.76, *p* < 0.05) **(C)**. On the other hand, for the whole study areas, there is little correlation between SST density changes and PV density changes, or between SST density changes and I/E changes for all three groups of mice (| *r*| < 0.5 or | *r*| ≈0.0) **(C)**.

Previous investigations on E/I balance were carried out in the neocortex and hippocampus (Kvitsiani et al., 2013, Moore and Wehr, 2013, Xue et al., 2014, Ferguson and Gao, 2018, Sohal and Rubenstein, 2019, Terstege and Epp, 2023). However, the limbic system is the first and foremost site for the progression of AD. Different from previous E/I balance studies, our study used AD and aging mouse models to explore the linear correlation between PV, SST, and E/I balance in brain regions including the S1BF, RSC, Pir, and BMA (Figure 14). Previous studies have concluded that PV rather than SST cells inhibit excitatory neurons (Xue et al., 2014). PV directly inhibits excitatory neurons and regulates E/I balance (Song et al., 2021), while SST also inhibits PV cells (Viollet et al., 2008) and has more complex effect on the network activity. Our experimental results show that PV cell density changes and I/E changes are moderately related between adult and aged WT mice across brain regions (Figure 14C), whereas there is little correlation between SST cell density changes and PV cell density changes, or between SST cell density changes and I/E changes. Thus, PV interneurons may more prominently regulate E/I balance.

The region of greatest relative inhibitory upregulation is S1BF-L5 in both adult 5xFAD and aged WT mice (Figures 14A,B, I/E changes). Meaningfully, in both adult 5xFAD and aged WT mice, S1BF-L5 is also the region with the highest growth rate of PV cells (Figures 14A,B, PV density changes). Our data imply that S1BF-L5 plays an important role in the E/I imbalance (Le Roux et al., 2008).

## Conclusion

The damaged brain regions vary across different mouse models. Utilizing the statistical differences in data of synapse, PV and SST expressing interneurons from the same brain region between distinct mice groups, we can conclude that amyloidosis may impair the S1BF and BMA, while senescence may harm all studied regions, including the S1BF, RSC, hippocampus, Pir, BMA, and DM. Previous research implied that aging coincides with the deterioration of olfactory function (Schubert et al., 2012).

We discovered that amyloidosis may disrupt the alignment of excitatory pre- and postsynaptic quantities. Amyloidosis may reduce the quantity of synapses and SST cells, but does not impact the counts of PV cells. By contrast, aging is linked to a decline in synapses, I/E, SST and PV cells (Figures 3, 4, 5, 7, 8). Aging is accompanied by a reduction in neuronal and synaptic connections (Morrison and Hof, 2007) and reduced neuroplasticity. These alterations can easily lead to E/I balance disorder. Aging mostly affects synapses and I/E ratios in Pir, BMA, and DM, but it primarily affects PV cells and SST cells in the hippocampus region.

## Data Availability

The original contributions presented in the study are included in the article/Supplementary material, further inquiries can be directed to the corresponding author.
